# Analysis of the Effect of Human-Machine Co-Driving Vehicle on Pedestrian Crossing Speed at Uncontrolled Mid-Block Road Sections: A VR-Based Case Study

**DOI:** 10.3390/ijerph19127208

**Published:** 2022-06-12

**Authors:** Kun Wang, Liang Xu, Han Jiang

**Affiliations:** 1 School of Transportation Science and Engineering, Beihang University, Beijing 102206, China; buaajh@buaa.edu.cn; 2 Beihang Hangzhou Innovation Institute Yuhang, Hangzhou 310023, China; xuliang@zfau.cn

**Keywords:** traffic safety, human-machine co-driving vehicle, pedestrian safety, crossing speed, time headway

## Abstract

The current study investigates the effects of speed and time headway of human-machine co-driving vehicles on pedestrian crossing speed at uncontrolled mid-block road sections. A VR-based simulation study is conducted to study pedestrian crossing behaviour when facing human-machine co-driving vehicles. A total of 30 college students are recruited, and each participant is required to complete 5 street-crossing simulator trials facing human-machine co-driving vehicles with varying time headway levels and speeds. The correlations and differences between demographic information, time headway, vehicle speed, and pedestrian crossing speed are analyzed. The results show that gender and pedestrian’s trust in human-machine co-driving vehicles are significantly correlated with pedestrian crossing speed. The pedestrian crossing speed increases with the increase in vehicle speed and decreases with the increase in vehicle time headway. In addition, the time headway has a stronger correlation with the pedestrian crossing speed than the vehicle speed. The findings will provide theoretical and methodological support for the formulation of pedestrian crossing control measures in the stage of human-machine co-driving.

## 1. Introduction

With the change in urban development concepts and the advancement of the green city process, residents have an increasing demand for walking. Pedestrians and motor vehicles are increasingly competing for the right of way on the roads with limited space and time resources, which leads to more road traffic accidents involving pedestrians. Globally, more than half of the 1.35 million people who died in road traffic crashes in 2016 were vulnerable road users, and approximately 23 percent of them were pedestrians [1]. The safety situation for pedestrians is even more serious in low- and middle-income countries. Although the total number of traffic accident deaths in China has decreased year by year, the number of pedestrian casualties caused by traffic accidents each year is still large [2]. The study of pedestrian traffic behavior has also become a crucial part of the field of pedestrian traffic system theory. Studies have shown that pedestrians are more susceptible to vehicle traffic when crossing the street, and collisions with motor vehicles are the most important cause of pedestrian casualties [3,4,5]. Among the characteristic variables of pedestrian crossing behavior, pedestrian crossing speed plays an important role in the design of pedestrian traffic facilities, traffic management, and control.

As the potential focus of future vehicles, the autonomous vehicle is generally regarded as one of the effective means to improve the reliability of road traffic, ensuring travel safety and reduce environmental pollution. However, with the development of autonomous vehicle technology, road traffic accidents involving autonomous vehicles have also increased. The popularity of autonomous vehicles will also lead to changes in the traffic behavior of other road traffic participants. Playing an important role in different development stages of the transportation systems, pedestrian safety in the autonomous driving environments is attracting more and more attention. On 18 March 2018, a woman in Arizona, USA, was injured by an Uber autonomous vehicle while crossing the street, which was the first autonomous vehicle accident involving pedestrians in the world. In addition, before reaching the degree of full automation of the vehicle, it is necessary to go through a long transition stage, that is, the ‘human-machine co-driving’ stage of L2/L3 according to the classification standard of SAE. At this stage, the autonomous vehicles are jointly controlled by drivers and automation systems, and the drivers are transitioning from operators to supervisors. Although drivers are no longer required to perform driving operations, they still need to monitor the road environment, driving situation, the status of the driving automation system and respond to the takeover request (TOR) of the driving automation system in time. At present, changes in the traffic environment, pedestrians’ inability to quickly and accurately determine the interaction of coming vehicles, and large differences in pedestrians’ trust in autonomous vehicles may lead to significant differences in the crossing speed of pedestrians. The effects of various factors on the speed of pedestrians crossing the street when facing human-machine co-driving vehicles are not yet clear. Therefore, it is undoubtedly of great theoretical and practical significance to carry out the research on pedestrian crossing speed when facing human-machine co-driving vehicles.

The literature review on pedestrian crossing speed is provided as follows.

## 2. Literature Review

As an important parameter in pedestrian traffic safety analysis, pedestrian crossing speed provides important information for road crossing facility layout and signal design. As early as the 1970s, Fruin [6] conducted a detailed study of the spatial characteristics of the pedestrian traffic flow field, behavioral characteristics, and pedestrian movement patterns, and found that men walked faster than women. Chandra and Bharti [7] also obtained a similar conclusion that pedestrian crossing speed at crosswalks followed a normal distribution. Moreover, they found the crossing speed of male pedestrians is higher than that of female pedestrians, and pedestrian crossing speed was higher in two-lane single-lane sections. Wilson and Grayson [8] found that the average walking speed was 1.32 m/s for men and 1.27 m/s for women.

The age of pedestrians also has a significant impact on the crossing speed. With the increase in age, the cognitive abilities of pedestrians, including visual perception, hearing sense, and reaction speed as well as their physical health will gradually decline, which affects their crossing judgment and execution [9,10,11]. Zhang and Xi [12] found that the crossing speed of the elderly at the signalized intersections is about 1.08 m/s. With the increase in the proportion of the elderly in the crossing crowd, the crossing speed gradually decreased. Thus the design speed of pedestrian crossing was recommended to be 0.86 m/s when there were more than 41% of pedestrians are elderly people. Gates et al. [13] found that pedestrians older than 65 (*n* = 326) were the slowest of all age groups, with an average walking speed of value 1.16 m/s and the 15th percentile walking speed of 0.92 m/s, while for younger pedestrians the speed is 1.48 m/s and 1.27 m/s, respectively.

Previous studies have shown that pedestrian crossing decisions are primarily dependent on the distance between the approaching vehicle and the pedestrian himself [9,14]. Yannis et al. [15] also found that the pedestrian crossing speed was significantly correlated to the distance between vehicles and pedestrians. When the vehicle approaches the pedestrian, the distance between the vehicle and pedestrian is smaller, the pedestrians have a stronger sense of urgency. As a consequence, they would prefer to give up crossing the street or crossing the street at a faster speed [16]. In addition, Pawar and Patil [17] revealed that pedestrian gap acceptance at mid-block Street crossings is highly influenced by the speed of the approaching vehicle. Liu and Tung [18] found that fast-approaching vehicles hastened the pedestrians to cross the street faster. Khatoon et al. [19] also found that the likelihood of crossing the road decreased when the pedestrian faced heavy vehicles (for example, buses and trucks) and increased when they faced a two-wheeler. Although many existing research results have found that vehicle types have a significant impact on pedestrian crossing behavior choice, they mainly focused on vehicle size and kinematic characteristics. Kadali and Vedagiri [20] found that the average pedestrian crossing speeds were 1.16 m/s, 1.25 m/s, 1.22 m/s, and 1.30 m/s for a 2-wheeler, auto-rickshaws, cars, heavy vehicles, respectively. This revealed that pedestrians were more likely to cross the road faster when facing heavy vehicles than when facing other types of vehicles.

At present, there are relatively few studies on the impact of autonomous vehicles on pedestrian crossing behavior and no consensus has been reached. Rodriguez Palmeiro et al. [21] explored the impact of autonomous vehicles on pedestrian crossing decisions by attaching autonomous vehicle logos to the body. Nuñez Velasco et al. [22] and Dey et al. [23] found that whether a vehicle is autonomous would affect pedestrians’ risk perception of the external environment, but there was no significant difference in their willingness to cross the street. However, Lundgren et al. [24] found that pedestrians’ willingness to cross the street was significantly reduced when drivers had no eye contact with pedestrians.

In summary, the researchers in the field of pedestrian crossing speed have carried out a large number of research works and abundant research results have been obtained. However, most studies were carried out according to the traditional traffic environment, while there were only a few studies on pedestrian crossing speed characteristics in the traffic environment of human-machine co-driving vehicles. Meanwhile, the effect of human-machine co-driving vehicle on pedestrian crossing speed is not clear. Therefore, through the pedestrian crossing simulation experiment in the traffic environment of human-machine co-driving vehicles at uncontrolled mid-block road sections, pedestrian crossing speeds facing human-machine co-driving vehicles with different time headways and vehicle speeds are collected. The effects of speed and time headway of human-machine co-driving vehicles on pedestrian crossing speed at road sections are explored. In addition, a questionnaire on the trust degree of human-machine co-driving vehicles is introduced into this study of pedestrian crossing speed. This work provides new insights and support to the design of pedestrian crossing control measures in the coming human-machine co-driving era.

## 3. Methods

### 3.1. Participants

A total of 32 college students from Beihang University and Zhejiang University were recruited to participate in the experiment. The recruitment criteria were as follows: (a) being in good health and not suffering from virtual reality sickness (as assessed by a trial test), and (b) no visual impairment such as myopia or astigmatism. A total of 2 participants were excluded due to motion sickness and failure to complete the test as required, and 30 experimental participants were ultimately obtained. The average age of the participants was 25.0 years (S.D. = 1.51). Of the 30 participants, 21 (70% of the total) were male. To ensure that the participants had a good mental state, they were forbidden to drink stimulant drinks such as tea or coffee before the experiment in order to reduce the influence of other factors on the subjects.

The sample size is very critical for an experiment. In this study, G*Power3.1.7 software (Franz Faul, Universität Kiel, Germany) is used to estimate the sample size [25,26]. The calculated result shows that power of 0.99 (α = 0.01) can be achieved when the sample size is 27. This implies that the sample size in our study is sufficient and can obtain reliable findings for the questions to be investigated.

### 3.2. Apparatus

**Figure 1:**
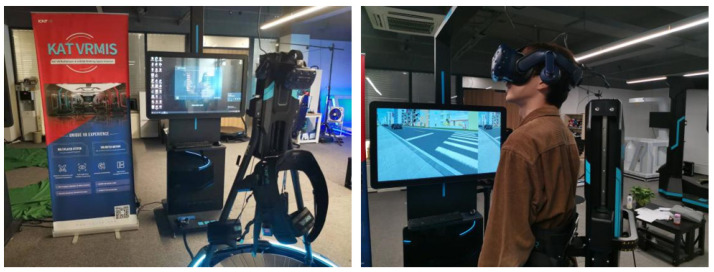
Walking simulation apparatus.

Two apparatus were used in this study, including Scanner Studio 1.6 software and walking simulation apparatus (KAT Walk Mini S system, Tokyo, Japan), as shown in Figure 1.

The simulated street crossing scenarios were customized using Scanner Studio 1.6 software, which is usually used to simulate driving scenarios in traffic behaviour research [25,27]. In addition, the vehicle’s inherent attributes (appearance) and traffic characteristics (speed and headway) can be also defined by the software. In the simulated street crossing scenarios, pedestrian crossing behavior data, including speed and location, was recorded at a frequency of 100 Hz.

The walking simulation apparatus (KAT Walk Mini S system) was used to simulate participants’ street crossing in Figure 1. It includes three parts: a universal mobile platform, VR, and a control center, which can realize 1:1 linear input, squat, run, walk, and jump without restraint. The apparatus has a resolution of more than 32,768 × 32,768 pixels that can accurately capture more than 10 moving point tracks. At the same time, the platform is equipped with an open software development kit (SDK), which can realize communication and interaction with third-party software data to extract the user’s real-time behavioral data.

In this study, we used Scanner Studio 1.6 software to construct and simulate crossing scenarios and then delivered it into VR by using a communication module. The participants were required to wear VR to complete street crossing experiments on walking simulation apparatus. Then, the walking simulation apparatus transferred the behaviour data back to the simulation software for recording.

### 3.3. Scenarios

**Figure 2:**
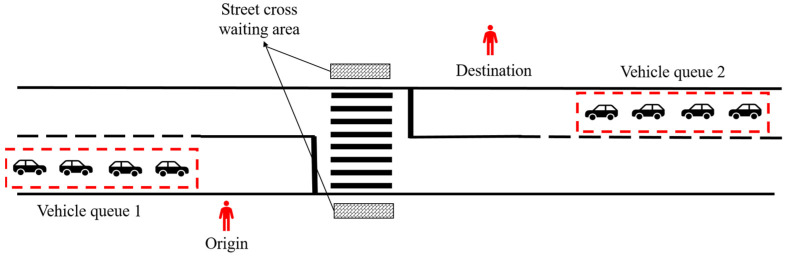
Schematic representation of the street crossing scenario.

The experiment had a 5 (time headway levels) × 5 (vehicle speeds) within-participants repeated measures design. The street crossing scenario was simulated on a section of road in a daytime environment. The experimental scene included five simulated roads with the same line shape, and pedestrian crossing facilities were set at the same position on the road section, as shown in Figure 2. According to the results of the existing findings, 5 simulated roads were loaded with five different kinds of time headway, which were 3 s, 4 s, 5 s, 6 s, and 7 s, respectively [28,29,30]. Each simulated road had a two-lane road with a single lane in each direction marked with single white edge lines and dotted yellow centre lines. The width of each lane was 3.75 m, and there were sidewalks on both sides of the road with a width of 0.5 m. The road sections are set with crossing scenes and no signal control. Each simulated road includes 5 vehicle queues, which were loaded with five different kinds of vehicle speeds, which were 20 km/h, 30 km/h, 40 km/h, 50 km/h, and 60 km/h. Furthermore, 4 human-machine co-driving vehicles were successively set in each vehicle queue. To eliminate the effect of time headway sequence, we constructed a Latin square to provide each participant with a random sequence of trials. Similarly, to mitigate the effect of human-machine co-driving vehicle speed sequence in each simulator trial, 5 vehicle queues with different vehicle speeds were randomly combined.

### 3.4. Experimental Procedure and Data Collection

Each participant participated in 5 trials. Participants had a break of 5~10 min between 2 adjacent trials to avoid the problem of fatigue. The experimental procedures are described as follows.

(1) Participants were briefed about the purpose of the study and given precautions. All of the participants were asked to sign an informed consent form. They understood their rights as participants, which were voluntary, and a monetary reward was given for completion. All of the participants were told that some of them might experience virtual reality sickness, characterized by dizziness, nausea, and vomiting. If any discomfort happens during the experiment, he or she could immediately stop the experiment and return to feeling normal after a short rest. For these participants, a certain compensation would be paid according to the proportion of time spent;
(2) Participants were introduced to the operation method of the apparatus and were required to perform a practice walk for 5~10 min to familiarize themselves with the operation of the apparatus. In addition, they could adjust their walking styles to the simulated vehicles and surrounding environment. The scenario used for the practice walk was not identical to that used in the main experiment;
(3) Participants were required to walk from the starting point to the street crossing waiting area. Then, when the first vehicle in the queue passed through the pedestrian’s position, the participant was required to choose an appropriate time to complete a street crossing mission according to the situation and walk to the destination. In this process, they should ensure that they would not be hit by vehicles;
(4) When the participant had completed the street crossing mission, he or she was asked to prepare for the next set of missions, which was to return to the starting point following the same requirements described above. There were three chances for each street crossing mission, and if there is a collision or a failed crossing, the trial would be restarted;
(5) Participants repeated steps (3) and (4) until all 5 trials were completed. Finally, a demographic information questionnaire was also required for each pedestrian. Information was collected regarding each participant’s age, gender, education, the degree of understanding of human-machine co-driving vehicles, the level of trust in human-machine co-driving vehicles, and the overall view of human-machine co-driving vehicles. In addition, participant crossing speed was collected.

## 4. Results

### 4.1. Analysis of the Influence of Personal Attributes on Street Crossing Speed

#### 4.1.1. Gender

The results of the independent *t*-test analysis in Table 1 indicate that there is a significant difference in the crossing speed of male and female pedestrians (*t* = −9.735, *p* < 0.001) when facing a human-machine co-driving vehicle. The average crossing speed of female pedestrians is 1.291 m/s, which is significantly lower than that of male pedestrians (1.489 m/s).

#### 4.1.2. Trust in Human-Machine Co-Driving Vehicle

Pedestrians’ trust level in human-machine co-driving vehicles is divided into 3 groups: distrust, general, and trust. Analysis of partial correlation controlled with time headway and vehicle speed is used to explore the relationship between trust in human-machine co-driving vehicles and pedestrian crossing speed. Table 2 indicates that there is a significant correlation between trust in human-machine co-driving vehicles and pedestrian crossing speed (r = −0.175, *p* < 0.001). The result shows that with the increase in pedestrians’ trust in human-machine co-driving vehicles, the pedestrian crossing speed gradually decreases.

ANOVA analysis is used to further explore the difference in pedestrian crossing speed with different trust levels in human-machine co-driving vehicles. The results of the homogeneity of variance show that the significant *p*-value of the recognition time is 0.078, which passes the variance homogeneity test. The least significant difference test (LSD) is also used for analysis. Table 3 shows that there are significant differences in pedestrian crossing speed with different trust levels in human-machine co-driving vehicles (F = 15.352, *p* < 0.001). When facing human-machine co-driving vehicles, the crossing speed of pedestrians who do not trust the safety of human-machine co-driving vehicles has a significantly higher crossing speed than that of those pedestrians who trust the safety of human-machine co-driving vehicles. However, there is no significant difference in crossing speed between the general trust group and the trust group.

### 4.2. Analysis of the Influence of Time Headway and Vehicle Speed on Street Crossing Speed

**Figure 3:**
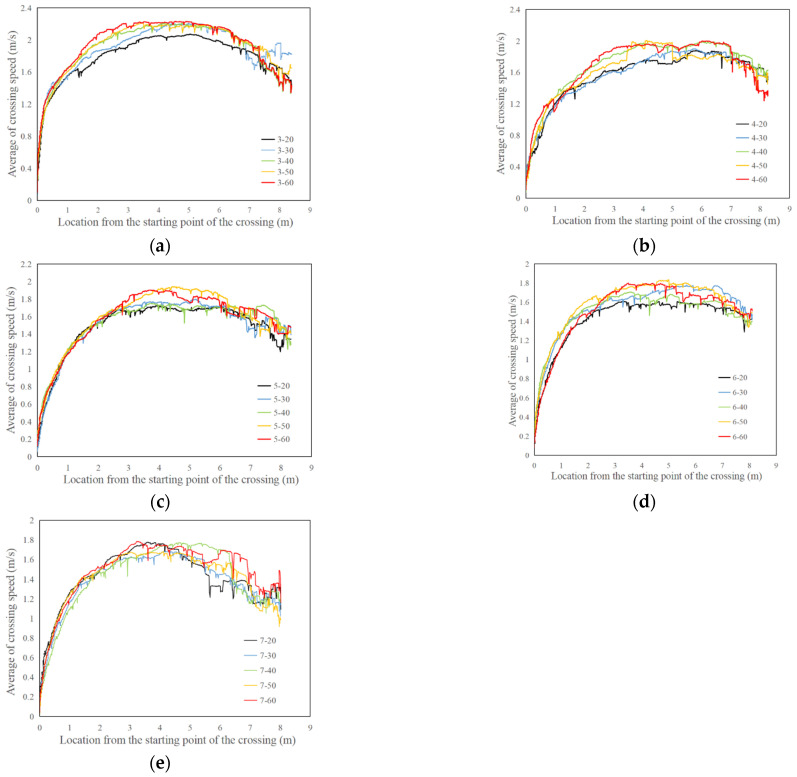
The continuous distribution of average crossing speed along the crosswalk. (**a**) Time headway = 3 s. (**b**) Time headway = 4 s. (**c**) Time headway = 5 s. (**d**) Time headway = 6 s. (**e**) Time headway = 7 s.

**Figure 4:**
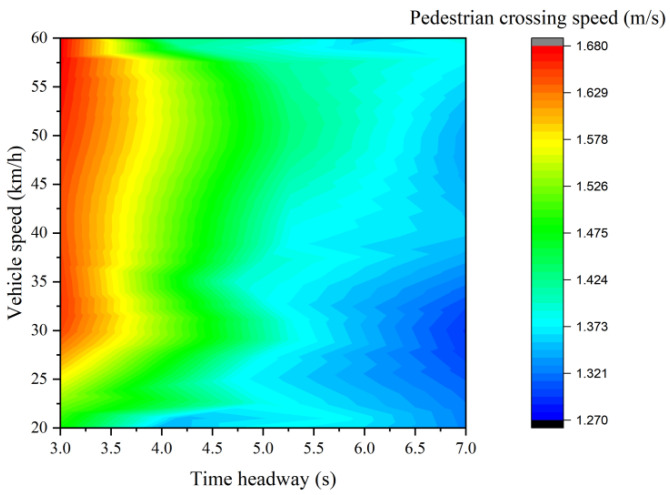
Variation in pedestrian crossing speed with vehicle time headway and vehicle speed.

Figure 3 shows the continuous distribution of average crossing speed along the crosswalk. The results show that the average pedestrian crossing speed increased at first and then decreased. Morevover, the average pedestrian crossing speed reaches its maximum value in the middle of the road crosswalk. In addition, the variation in pedestrian crossing speed with time headway and vehicle speed was explored in Figure 4. The results indicate that the pedestrian crossing speed increases with the increase in human-machine co-driving vehicle speed and decreases with the increase in vehicle time headway.

An analysis of partial correlation is used to explore the correlation between the pedestrian crossing speed and time headway or vehicle speed. Table 4 shows that when the vehicle speed variable is controlled, there is a strong significant negative correlation between the time headway and the pedestrian street crossing speed (r = −0.339, *p* < 0.001). Similarly, Table 5 shows that when the time headway variable is controlled, there is a significant positive correlation between the vehicle speed and the pedestrian street crossing speed (r = 0.093, *p* = 0.011).

ANOVA analysis is used to further explore the difference in pedestrian crossing speed when facing human-machine co-driving vehicles with different speeds and time headways. The results of the homogeneity of variance showed that the significant *p*-value of the vehicle speeds and time headway are 0.391 and 0.814, which passes the variance homogeneity test. The ANOVA analysis results show that the pedestrian crossing speed in different human-machine co-driving vehicle speeds has a significant difference in 90% confidence interval (F = 1.982, *p* = 0.095), and the pedestrian crossing speed in different time headways has a significant difference in 99% of the confidence intervals (F = 31.304, *p* ≤ 0.001).

In addition, the least significant difference test (LSD) is used to carry out multiple comparison tests on pedestrian crossing speeds facing human-machine co-driving vehicles with different speeds and headways. Table 6 shows that there are significant differences in pedestrian crossing speed when the vehicle speed is 20 km/h, 50 km/h, and 60 km/h. With the increase in the vehicle speed, the pedestrian crossing speed also increases significantly. However, there is no significant difference in the pedestrian crossing speed among other vehicle speeds. Table 7 also shows that there are significant differences in pedestrian crossing speed under different time headway conditions, except for the time headway of 4 s and 5 s, 6 s, and 7 s. With the increase in the vehicle time headway, the pedestrian crossing speed decreases.

## 5. Discussion

In this study, the effects of human-machine co-driving vehicles on pedestrian crossing performance, especially street crossing speed, are investigated, taking the effect of vehicle speed and time headway of human-machine co-driving vehicles into account. In addition, the influence of personal attributes, including gender and pedestrians’ trust in human-machine co-driving vehicles, on their street crossing speed are also explored.

Gender is a significant variable that affects pedestrian crossing speed when facing human-machine co-driving vehicles. The average street crossing speed of males is significantly higher than that of females, which is consistent with the previous research results on the crossing characteristics of male and female pedestrians in the traditional street crossing environment [31,32,33,34]. Most of the male pedestrians were in better physical and physiological conditions than female pedestrians, so the walking speeds of male pedestrians are generally faster than those of female pedestrians. In traffic behaviors, male pedestrians are more likely to make high-risk traffic decisions than females, which are manifested as faster crossing speeds and a higher incidence of high-risk crossing behaviors [35]. More attention should be paid to the traffic regulations and the improvement of safety awareness for the male pedestrian groups.

This study reveals that as pedestrians’ trust in human-machine co-driving vehicles decreases, their crossing speeds gradually increased. Some studies have identified trust as a relevant antecedent of risk perception and found that higher levels of trust reduce the level of risk perception over time [36,37,38]. Therefore, pedestrians with lower trust levels in human-machine co-driving vehicles often perceive a greater risk of crossing the street when facing human-machine co-driving vehicles. When pedestrians face persistent high-risk crossing scenarios, they would take more aggressive actions (faster crossing speeds, etc.) to compensate for the risks posed by such scenarios. Therefore, it is important to promote the trust of pedestrians by popularizing the knowledge of autonomous driving, so as to make their crossing behaviors more reliable and stable. In addition, a reasonable external human-machine interface (eHMI) of autonomous vehicles will help pedestrians understand the intentions of autonomous vehicles and thus ensure the safety of the street crossing.

There is a significant positive correlation between vehicle speed and pedestrian street crossing speed (r = 0.093, *p* = 0.011). With the increase in vehicle speed, the pedestrian crossing speed significantly increases, which is consistent with the findings of existing research [18,39]. Pedestrians crossing the street tend to be more nervous when facing a faster human-machine co-driving vehicle, which also makes them more cautious about their crossing behavior. For example, they may increase their crossing speed to ensure their safety. Liu and Tung [18] also found that fast-approaching vehicles stress them and may force them to cross the road faster. The existing research shows that pedestrians’ inaccurate estimate of vehicle speed would increase their risk of incorrectly deciding to cross when it is not safe to do so [39]. Therefore, safety education and speed estimating training exercises would improve the pedestrian performance at speed estimation, which in turn ensures their crossing safety.

There is a significant negative correlation between headway and pedestrian crossing speed (r = −0.339, *p* ≤ 0.001). Liu and Tung [18] also found that as the time interval increased, pedestrians were more likely to cross the road at a normal speed. When the headway of the human-machine co-driving vehicle increases, pedestrians will have more time to complete the entire crossing behavior, so they will choose a more comfortable speed to complete the entire crossing process on the premise of ensuring safety.

In addition, this work indicates that the time headway (r = −0.339, *p* ≤ 0.001) has a stronger correlation with the mean pedestrian crossing speed than the human-machine co-driving vehicle speed (r = 0.093, *p* = 0.011). Oxley et al. [9] found that when the headway was larger, pedestrians tended to make more aggressive crossing decisions (pressing ‘yes’ to indicate they would cross the road), ignoring the increasing vehicle speed. This may be because distance information is easier to be processed than vehicle speed and the underestimation bias increases as the vehicle speed increases. Greater underestimation bias would occur when a vehicle speed is higher, meaning that the difference between the two-vehicle speeds perceived by pedestrians is insignificant [39].

The present study also has some limitations. This study only considers pedestrian crossing scenarios facing human-machine co-driving vehicles on the road sections. The traffic flow environment and road attributes at intersections are different from those at road sections, which will affect pedestrian crossing behavior. The next step of work is to enrich the street crossing scenarios and explore the differences in different pedestrian crossing behaviors facing human-machine co-driving vehicles in different road scenarios. Meanwhile, the participants of this study are mainly young pedestrians, and the influence of different age levels has not been considered. Pedestrians’ crossing speed could be affected by pedestrians’ age, the study on behaviors of people at other ages is required to evaluate such effects. In addition, in order to increase the engineering application value of the research results, real vehicle experiments are expected to be carried out to validate the relevant results.

## 6. Conclusions

A VR-based simulation experiment is constructed to explore the characteristics of pedestrian street crossing speed when facing human-machine co-driving vehicles with different time headways and speeds. In addition, the influence of personal attributes, such as gender and trust level in human-machine co-driving vehicles, on their street crossing speed are also explored. 

More specifically, the following results were obtained: (a) the crossing speed when facing human-machine co-driving vehicles of female pedestrians is 1.291 m/s, which is significantly lower than that of male pedestrians (1.489 m/s), and (b) there is a significant negative correlation between pedestrian trust in pedestrians’ trust in human-machine co-driving vehicles and their crossing speeds, namely, with the increase in pedestrians’ trust in human-machine co-driving vehicles, the pedestrian crossing speed gradually decreases, and (c) the pedestrian crossing speed has a significant negative correlation with the time headway and a significant positive correlation with the vehicle speed. Moreover, the time headway (r = −0.339, *p* ≤ 0.001) has a stronger correlation with the average pedestrian crossing speed than the human-machine co-driving vehicle speed (r = 0.093, *p* = 0.011). These findings can provide theoretical and methodological support for the formulation of pedestrian crossing control measures at the stage of human-machine co-driving.

## Figures and Tables

**Table 1:** Difference analysis of gender on pedestrian crossing speed.

Variable	Range	*n*	Mean	S.D.	*t*	Sig.
Gender	Female	9	1.291	0.248	−9.735	<0.001
	Male	21	1.489	0.259		

**Table 2:** Analysis of the partial correlation between trust in human-machine co-driving vehicles and pedestrian crossing speed.

Control Variable	Variable	Trust in Human-Machine Co-Driving Vehicle	Pedestrian Crossing Speed
Time headway & vehicle speed	Trust in human-machine co-driving vehicle	1.000	−0.175 ***
	pedestrian crossing speed	−0.175 ***	1.000

*** *p* ≤ 0.001.

**Table 3:** LSD multiple comparisons of trust in human-machine co-driving vehicle.

Trust Level in Human-Machine Co-Driving Vehicle (I)	Trust Level in Human-Machine Co-Driving Vehicle (J)	Mean Difference (I-J)	Sig.	95% Confidence Interval	
				Lower	Upper
Distrust	General	0.131 ***	<0.001	0.081	0.181
	Trust	0.140 ***	<0.001	0.084	0.196
General	Trust	0.009	0.703	−0.036	0.054

*** *p* ≤ 0.001.

**Table 4:** Analysis result of the partial correlation between time headway and pedestrian crossing speed.

Control Variable	Variable	Time Headway	Pedestrian Crossing Speed
Vehicle speed	Time headway	1.000	−0.339 ***
	Pedestrian crossing speed	−0.339 ***	1.000

*** *p* ≤ 0.001.

**Table 5:** Analysis result of the partial correlation between vehicle speed and pedestrian crossing speed.

Control Variable	Variable	Vehicle Speed	Pedestrian Crossing Speed
Time headway	Vehicle speed	1.000	0.093 ***
	Pedestrian crossing speed	0.093 ***	1.000

*** *p* ≤ 0.001.

**Table 6:** Multiple comparisons of the effect of vehicle speeds on pedestrian crossing speed.

Vehicle Speed (I)	Vehicle Speed (J)	Mean Difference (I-J)	Sig.	95% Confidence Interval	
				Lower	Upper
20	30	−0.0548	0.079	−0.116	0.0064
	40	−0.0440	0.159	−0.105	0.0173
	50	−0.0784	0.012	−0.140	−0.0172
	60	−0.0721	0.021	−0.133	−0.0109
30	40	0.0108	0.729	−0.0504	0.0720
	50	−0.0236	0.449	−0.0849	0.0376
	60	−0.0174	0.578	−0.0786	0.0439
40	50	−0.0344	0.270	−0.0957	0.0268
	60	−0.0282	0.367	−0.0894	0.0331
50	60	0.0063	0.841	−0.0550	0.0675

**Table 7:** Multiple comparisons of the effect of vehicle time headway on pedestrian crossing speed.

Time Headway (I)	Time Headway (J)	Mean Difference (I-J)	Sig.	95% Confidence Interval	
				Lower	Upper
3	4.000	0.194	<0.001	0.137	0.251
	5.000	0.215	<0.001	0.158	0.272
	6.000	0.277	<0.001	0.220	0.334
	7.000	0.282	<0.001	0.225	0.339
4	5.000	0.021	0.476	−0.036	0.078
	6.000	0.083	0.004	0.026	0.140
	7.000	0.088	0.003	0.031	0.145
5	6.000	0.062	0.032	0.005	0.119
	7.000	0.067	0.021	0.010	0.124
6	7.000	0.005	0.865	−0.052	0.062

## Data Availability

Data will be made available on reasonable request from the corresponding author.

## References

[1] World Health Organization (2018). Global Status Report on Road Safety 2018: Summary.

[2] National Bureau of Statistics of China (2021). China Statistics Yearbook 2021.

[3] World Health Organization (2015). World Health Statistics 2014.

[4] Fu L., Zou N. (2016). The influence of pedestrian countdown signals on children’s crossing behavior at school intersections. Accid. Anal. Prev..

[5] Zhang W.H., Wang K., Wang L., Feng Z.X., Du Y.J. (2016). Exploring factors affecting pedestrians’ red-light running behaviors at intersections in China. Accid. Anal. Prev..

[6] Fruin J.J. (1971). Pedestrian Planning and Design.

[7] Chandra S., Bharti A.K. (2013). Speed Distribution Curves for Pedestrians During Walking and Crossing. Procedia Soc. Behav. Sci..

[8] Wilson D.G., Grayson G.B. (1980). Age-Related Differences in the Road Crossing Behaviour of Adult Pedes-Trians.

[9] Oxley J.A., Ihsen E., Fildes B.N., Charlton J., Day R.H. (2005). Crossing roads safely: An experimental study of age differences in gap selection by pedestrians. Accid. Anal. Prev..

[10] Lobjois R., Cavallo V. (2009). The effects of aging on street-crossing behavior: From estimation to actual crossing. Accid. Anal. Prev..

[11] Holland C., Hill R. (2010). Gender differences in factors predicting unsafe crossing decisions in adult pedes-trians across the lifespan: A simulation study. Accid. Anal. Prev..

[12] Zhang H.L., Xi B.S. (2021). Signalized intersection pedestrian crossing design speed and elderly pedestrian proportion relationship study. J. Transp. Syst. Eng. In..

[13] Gates T.J., Noyce D.A., Bill A.R., Andrea R. (2006). Recommended walking speeds for pedestrian clear-ance timing based on pedestrian characteristics. Transp. Res. Record J. Transp. Res. Board..

[14] Oxley J., Lenné M., Corben B. (2006). The effect of alcohol impairment on road-crossing behaviour. Transp. Res. Part F Traffic Psychol. Behav..

[15] Yannis G., Papadimitriou E., Theofilatos A. (2013). Pedestrian gap acceptance for mid-block street crossing. Transp. Plan. Technol..

[16] Jakym J., Attalla S., Kodsi S. (2013). Modeling of Pedestrian Mid-Block Crossing Speed with Respect to Ve-Hicle Gap Acceptance.

[17] Pawar D.S., Patil G.R. (2015). Pedestrian temporal and spatial gap acceptance at mid-block street crossing in developing world. J. Saf. Res..

[18] Liu Y.C., Tung Y.C. (2014). Risk analysis of pedestrians’ road-crossing decisions: Effects of age, time gap, time of day, and vehicle speed. Safety Sci..

[19] Khatoon M., Tiwari G., Chatterjee N. (2013). Impact of grade separator on pedestrian risk taking behavior. Accid. Anal. Prev..

[20] Kadali B.R., Vedagiri P. (2019). Evaluation of pedestrian crossing speed change patterns at unprotected mid-block crosswalks in India. J. Traffic Transp. Eng. English Ed..

[21] Palmeiro A.R., Van Der Kint S., Vissers L., Farah H., De Winter J.C.F., Hagenzieker M. (2018). Interaction between pedestrians and automated vehicles: A Wizard of Oz experiment. Transp. Res. Part F Traffic Psychol. Behav..

[22] Nuñez Velasco J.P., Farah H., van Arem B., Hagenzieker M.P. (2019). Studying pedestrians’ crossing be-havior when interacting with automated vehicles using virtual reality. Transp. Res. Part F Traffic Psychol. Behav..

[23] Dey D., Martens M., Eggen B., Terken J. (2019). Pedestrian road-crossing willingness as a function of ve-hicle automation, external appearance, and driving behaviour. Transp. Res. Part F Traffic Psychol. Behav..

[24] Lundgren V.M., Habibovic A., Andersson J. (2017). Will there be new communication needs when intro-ducing automated vehicles to the urban context?. Advances in Human Aspects of Transportation.

[25] Faul F., Erdfelder E., Lang A.-G., Buchner A. (2007). G*Power 3: A flexible statistical power analysis pro-gram for the social, behavioral, and biomedical sciences. Behav. Res. Meth..

[26] Chow S.C., Shao J., Wang H., Lokhnygina Y. (2017). Sample Size Calculations in Clinical Research Third Edition.

[27] Wang K., Zhang W., Feng Z., Yu H.Z., Wang C.J. (2021). Reasonable driving speed limits based on recognition time in a dynamic low-visibility environment related to fog—A driving simulator study. Accid. Anal. Prev..

[28] DiPietro C.M., King L.E. (1970). Pedestrian gap-acceptance. High. Res. Rec..

[29] Kadali B.R., Perumal V. (2012). Pedestrians’ Gap Acceptance Behavior at Mid Block Location. Int. J. Eng. Technol..

[30] Doric I., Frison A.K., Wintersberger P. A Novel Approach for Researching Crossing Behavior and Risk Acceptance: The Pedestrian Simulator. Proceedings of the 8th International Conference on Automotive User Interfaces and Interactive Vehicular Applications.

[31] Goh B.H., Subramaniam K., Wai Y.T., Mohamed A.A. (2012). Pedestrian crossing speed: The case of Ma-laysia. Int. J. Traffic Transp. Eng..

[32] Jain A., Gupta A., Rastogi R. (2014). Pedestrian Crossing Behaviour Analysis at Intersections. Int. J. Traffic Transp. Eng..

[33] Onelcin P., Alver Y. (2017). The crossing speed and safety margin of pedestrians at signalized intersections. Transp. Res. Procedia.

[34] Zafri N.M., Rony A.I., Adri N. (2019). Analysis of pedestrian crossing speed and waiting time at intersec-tions in Dhaka. Infrastructures.

[35] Holland C., Hill R. (2007). The effect of age, gender and driver status on pedestrians’ intentions to cross the road in risky situations. Accid. Anal. Prev..

[36] Pavlou P.A. (2003). Consumer acceptance of electronic commerce: Integrating trust and risk with the tech-nology acceptance model. Int. J. Electro. Comm..

[37] Kim D.J., Ferrin D.L., Rao H.R. (2008). A trust-based consumer decision-making model in electronic com-merce: The role of trust, perceived risk, and their antecedents. Decis. Support Syst..

[38] Choi J.K., Ji Y.G. (2015). Investigating the Importance of Trust on Adopting an Autonomous Vehicle. Int. J. Human-Computer Interact..

[39] Sun R., Zhuang X., Wu C., Zhao G., Zhang K. (2015). The estimation of vehicle speed and stopping dis-tance by pedestrians crossing streets in a naturalistic traffic environment. Transp. Res. Part F Traffic Psychol. Behav..

